# Supplementary figures and images for: Induction of tunnelling nanotube-like structures by influenza A viruses requires the onset of apoptosis
Source: PLoS Pathog. 2025 Jun 5;21(6):e1013191. doi: 10.1371/journal.ppat.1013191 (PMC12169559; doi:10.1371/journal.ppat.1013191)

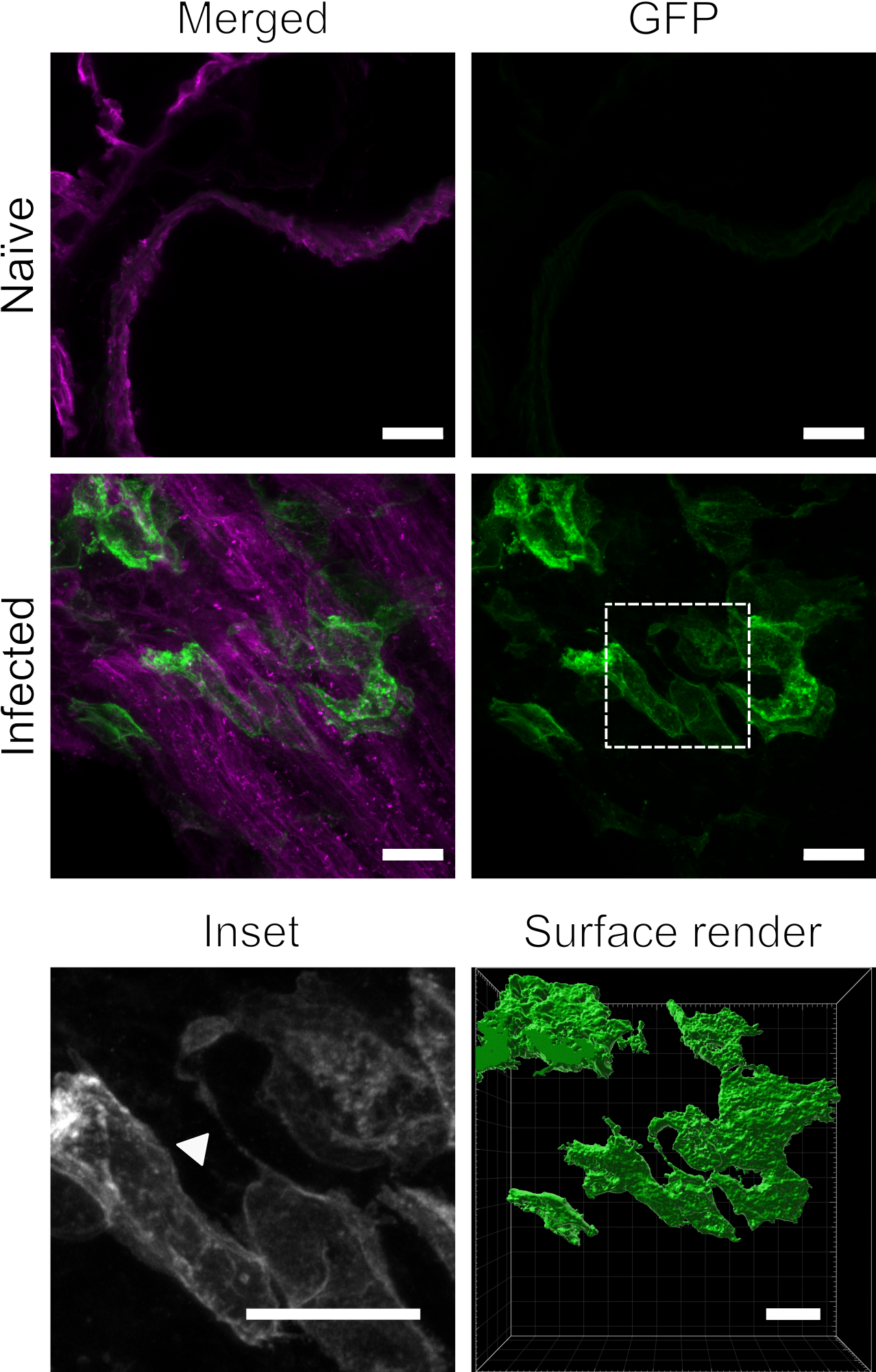

Supplement: S1 Fig — Maximum intensity projection of a TNT-like structure (TLS) within thick sectioned lung tissue connecting IAV infected cells. Magnified inset outlined in a white box is shown alongside a 3D render (bottom right panel). Cell membranes are labelled in tdTomato (magenta) for uninfected cells, and GFP (green) for infected cells. White arrow indicates the presence of a TLS. Scale bars = 20 µm. (TIFF) [file ppat.1013191.s001.tiff]

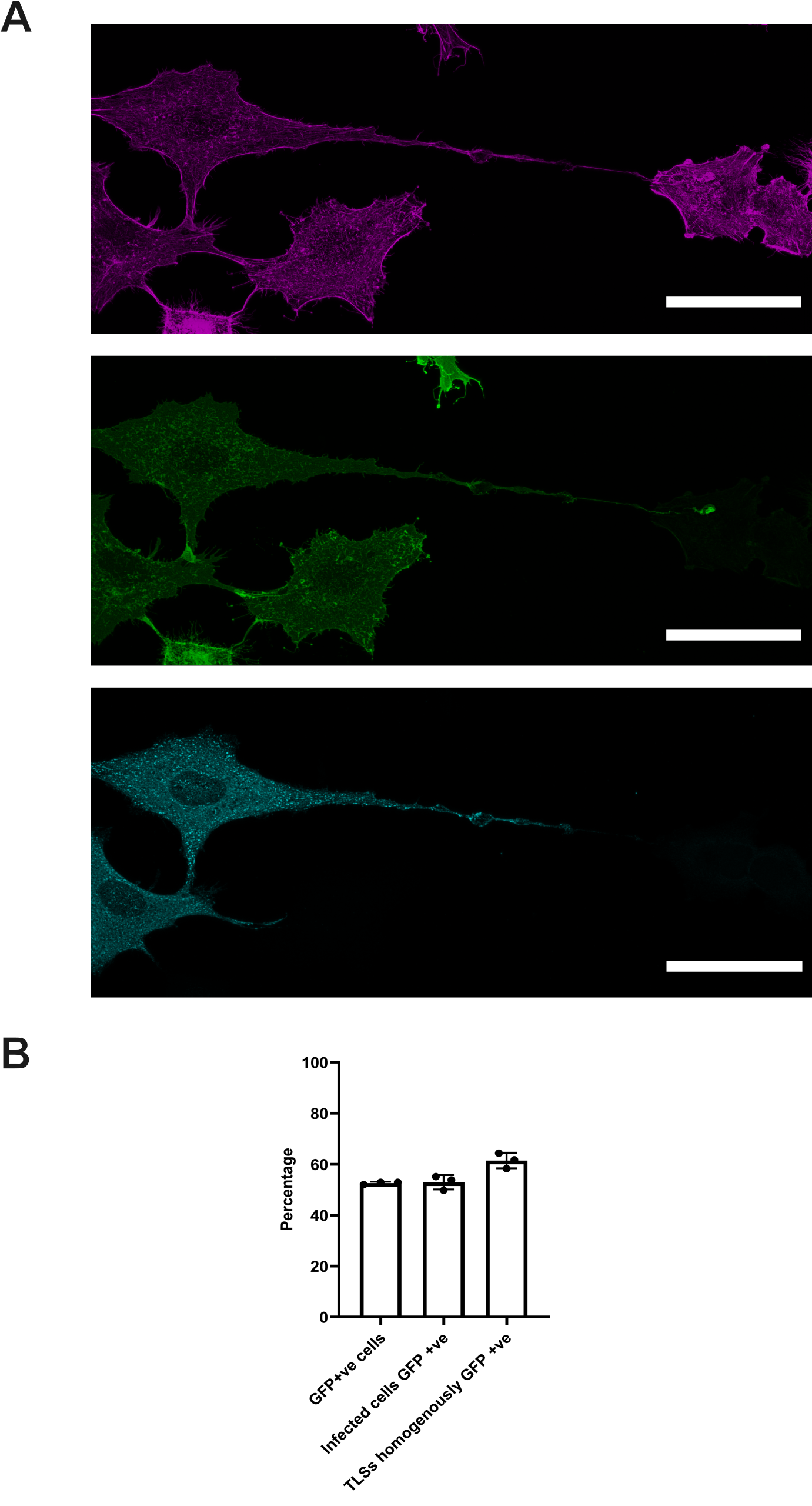

Supplement: S2 Fig — (A) Enlarged split channel images of A549 AcGFP1 cells cocultured with WT A549 cells at a ratio of 1:1 and infected at a low MOI. 16 hours post infection the cells were fixed and immunostained for NP. Nuclei (yellow), F-actin (magenta), AcGFP (green), NP (blue). Scale bar = 50 μm. (B) Following manual assessment of micrographs, the percentage of cells in coculture that were GFP positive (i.e., A549 AcGFP), GFP positive and infected and the percentage of TLS consisting of GFP labelled membrane throughout its length was determined. The means and standard deviations of three biological replicates are shown. (TIFF) [file ppat.1013191.s002.tiff]

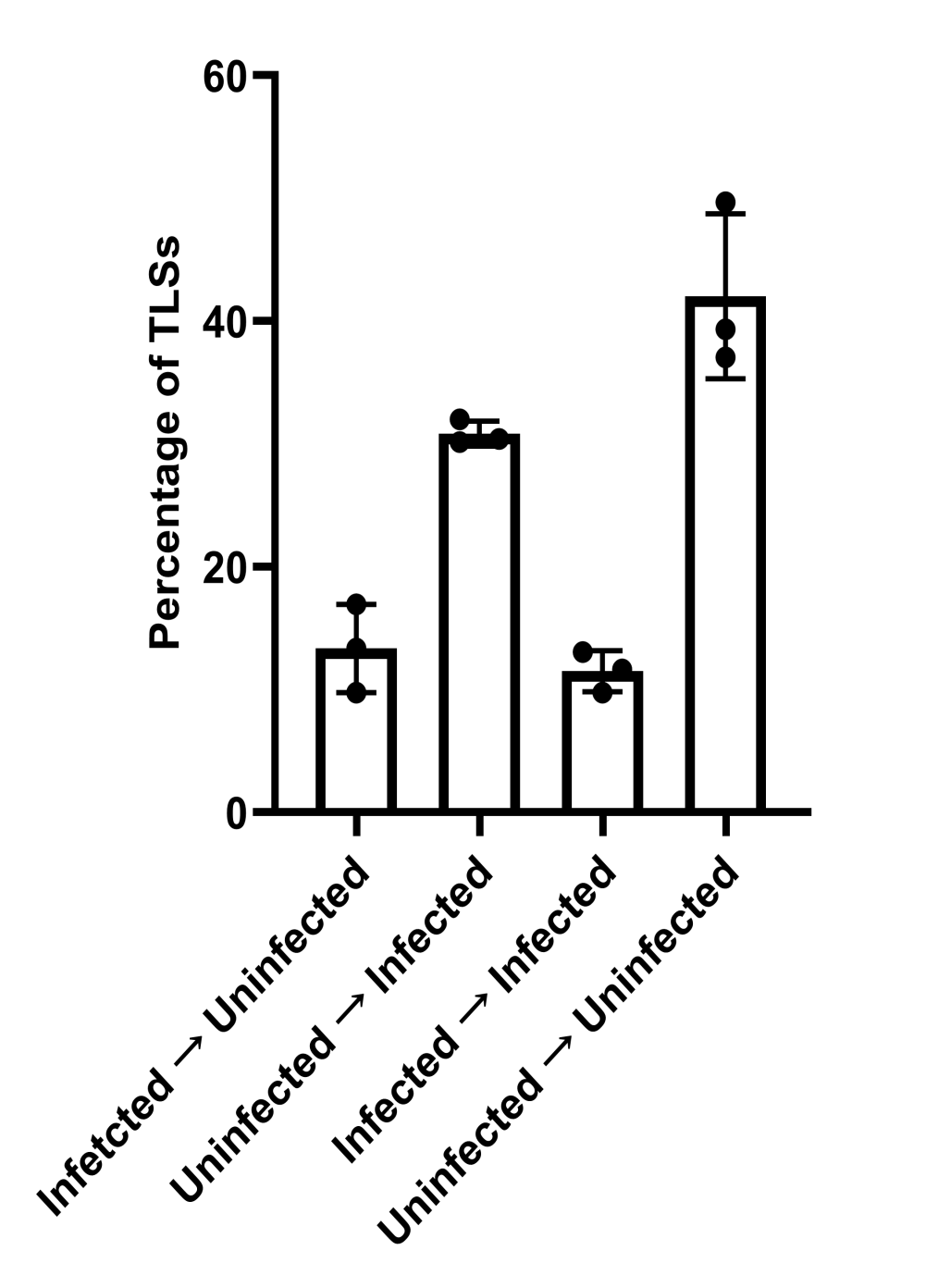

Supplement: S3 Fig — Percentage of TLSs connecting WT and AcGFP labelled A549 cells with both asymmetric and symmetric infection status. The means and standard deviations of 3 biological replicates are shown. (TIFF)\ [file ppat.1013191.s003.tiff]

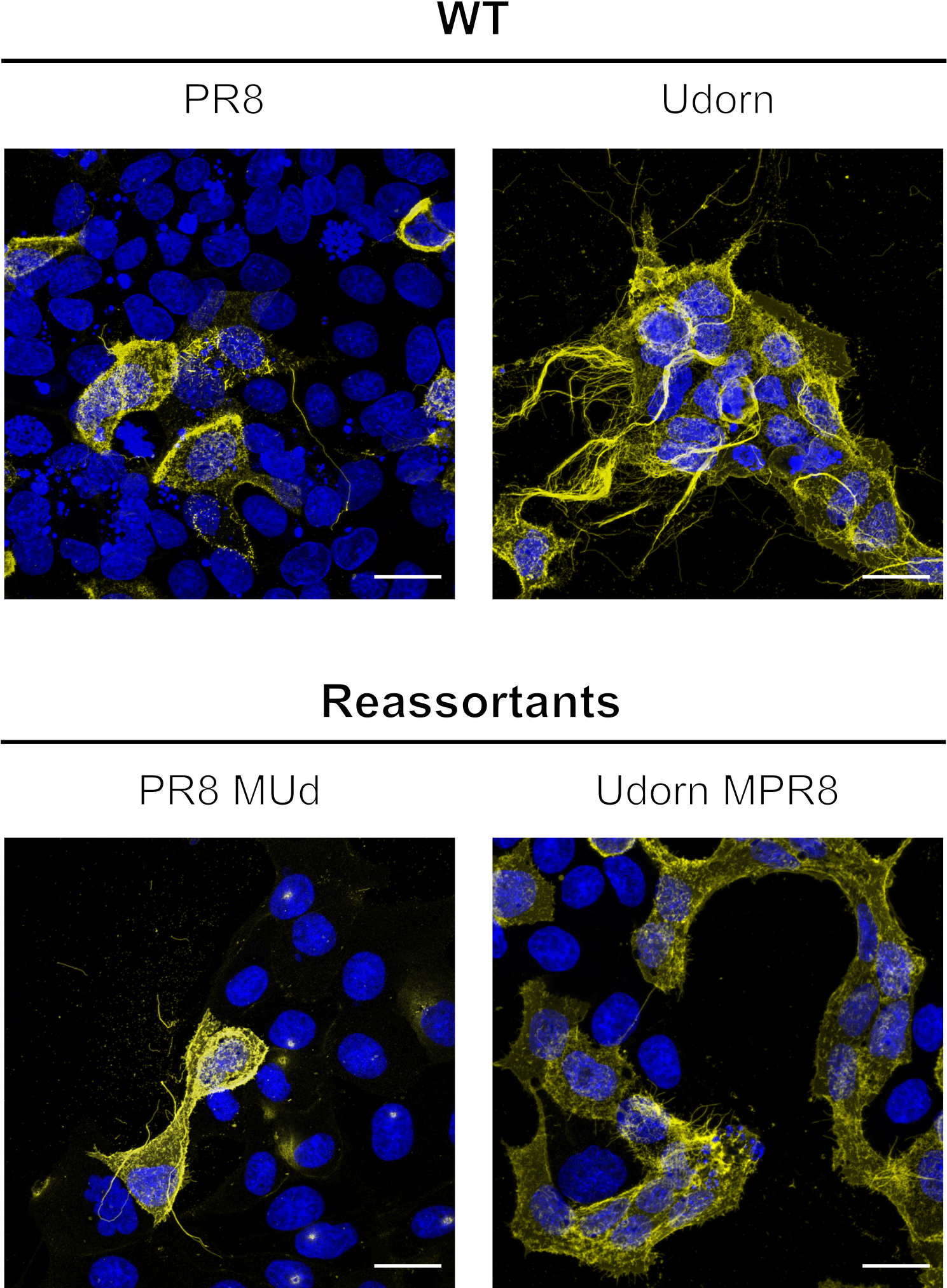

Supplement: S4 Fig — Maximum intensity projections of surface HA labelled MDCK cells, at 16 hours post infection with the IAV strains PR8 and Udorn (WT) or the segment 7 reassortant viruses Udorn MPR8 or PR8 MUd. DAPI (blue), HA (yellow). Scale bars = 20 μm. (TIFF) [file ppat.1013191.s004.tiff]

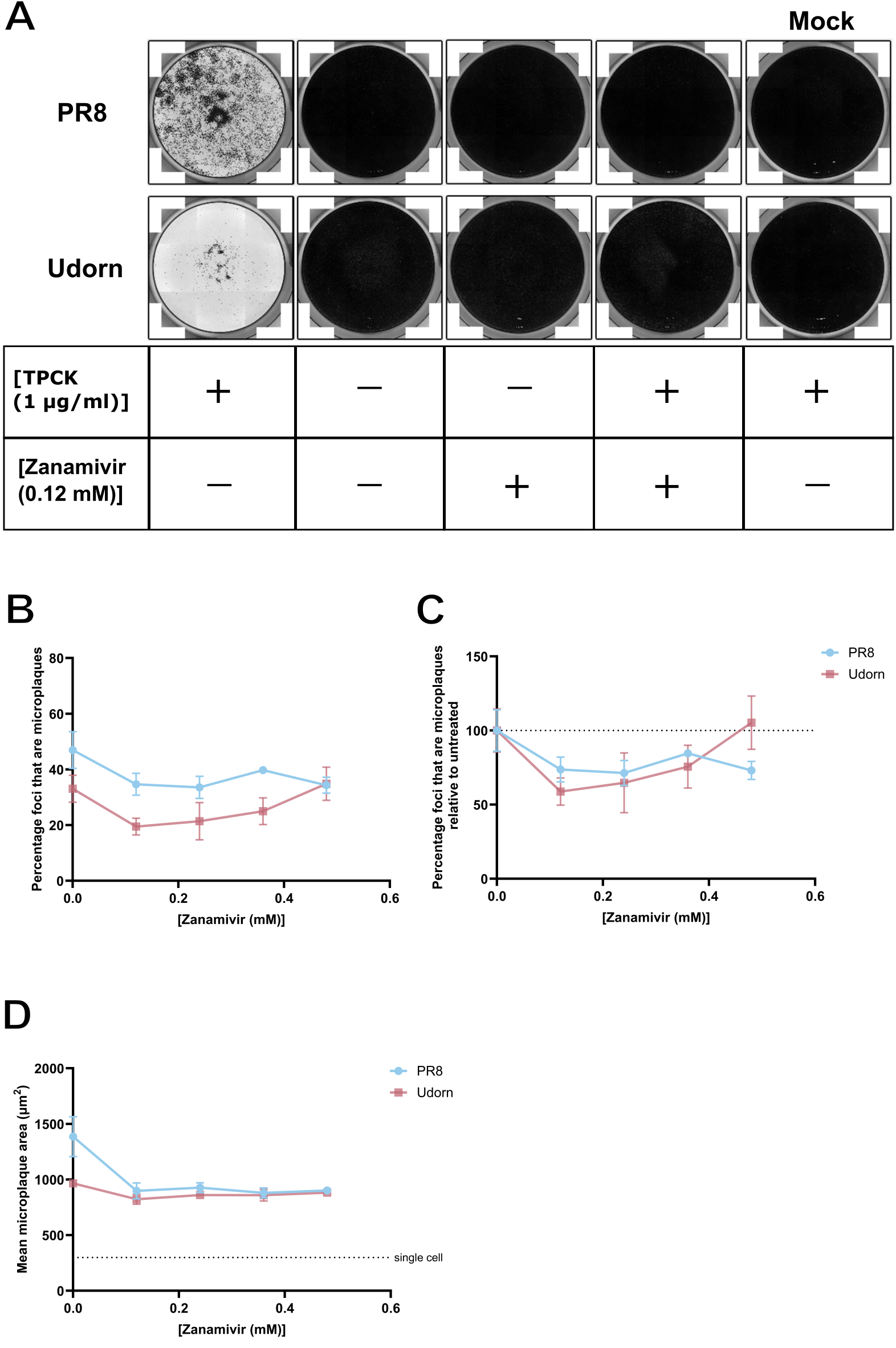

Supplement: S5 Fig — (A) Coomassie stained MDCK cells in 12 well plates, 48 hours post infection with the IAV strains PR8 or Udorn under microplaque assay conditions. The influence of Zanamivir (0.12 mM) or TPCK trypsin (1 µg/ml) was assessed by cytopathic effect (CPE). Images are representative of two biological replicates. (B) The percentage NP positive foci that form microplaques under increasing zanamivir concentrations. Differences between viruses at each concentration were tested for significance by Mann-Whitney test, and differences between concentrations were tested by Kruskal-Wallis test (n.s. p > 0.05). The means and standard deviations of three biological replicates are shown. The same data are shown in (C) with each virus normalised to its behaviour in the absence of zanamivir. (D) Mean microplaque area under increasing zanamivir concentrations, with a dashed line showing the approximate area of a single cell. The means and standard deviations of three biological replicates are shown. The significance of differences between viruses was determined by Kruskal-Wallis test (n.s. p > 0.05). (TIFF) [file ppat.1013191.s005.tiff]

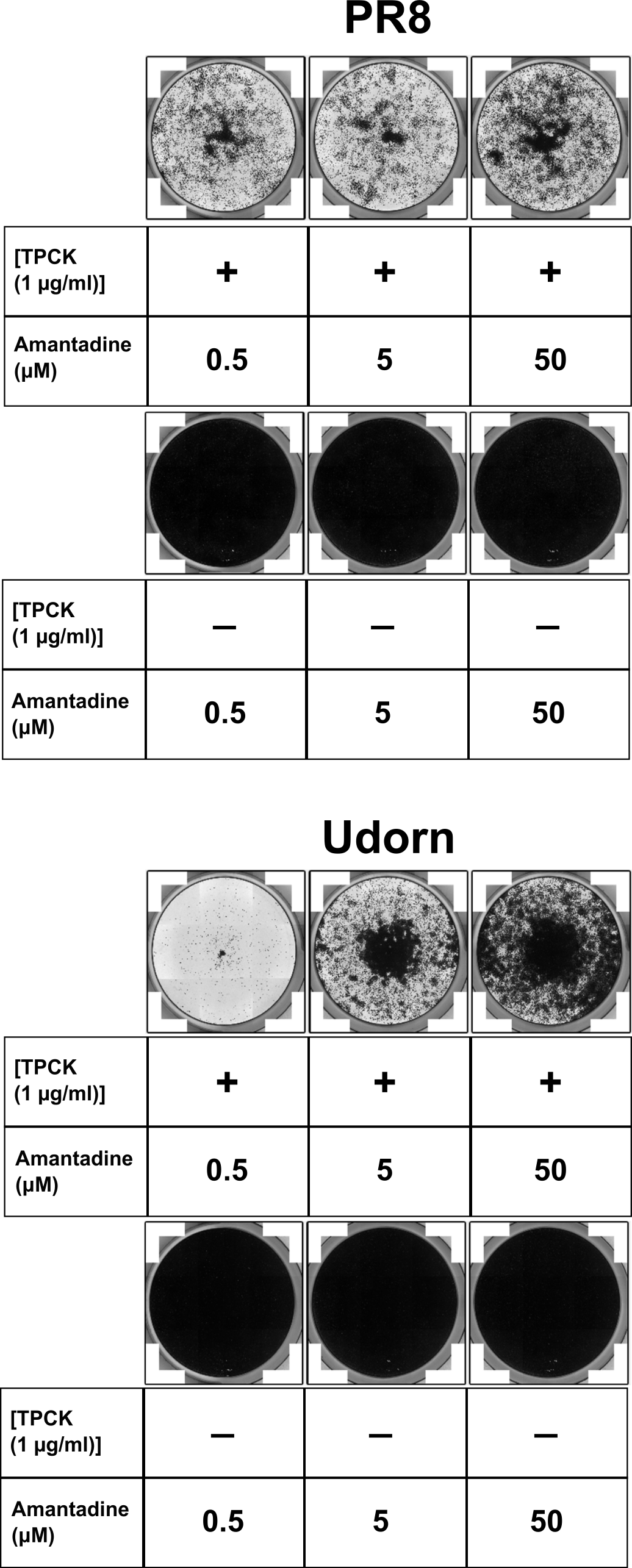

Supplement: S6 Fig — Coomassie stained MDCKs within a 12 well plate, 48 hours post infection with either PR8 or Udorn, in the presence (upper panel) and absence (lower panel) of TPCK trypsin at increasing amantadine concentrations. Images are representative of two biological replicates. (TIFF) [file ppat.1013191.s006.tiff]

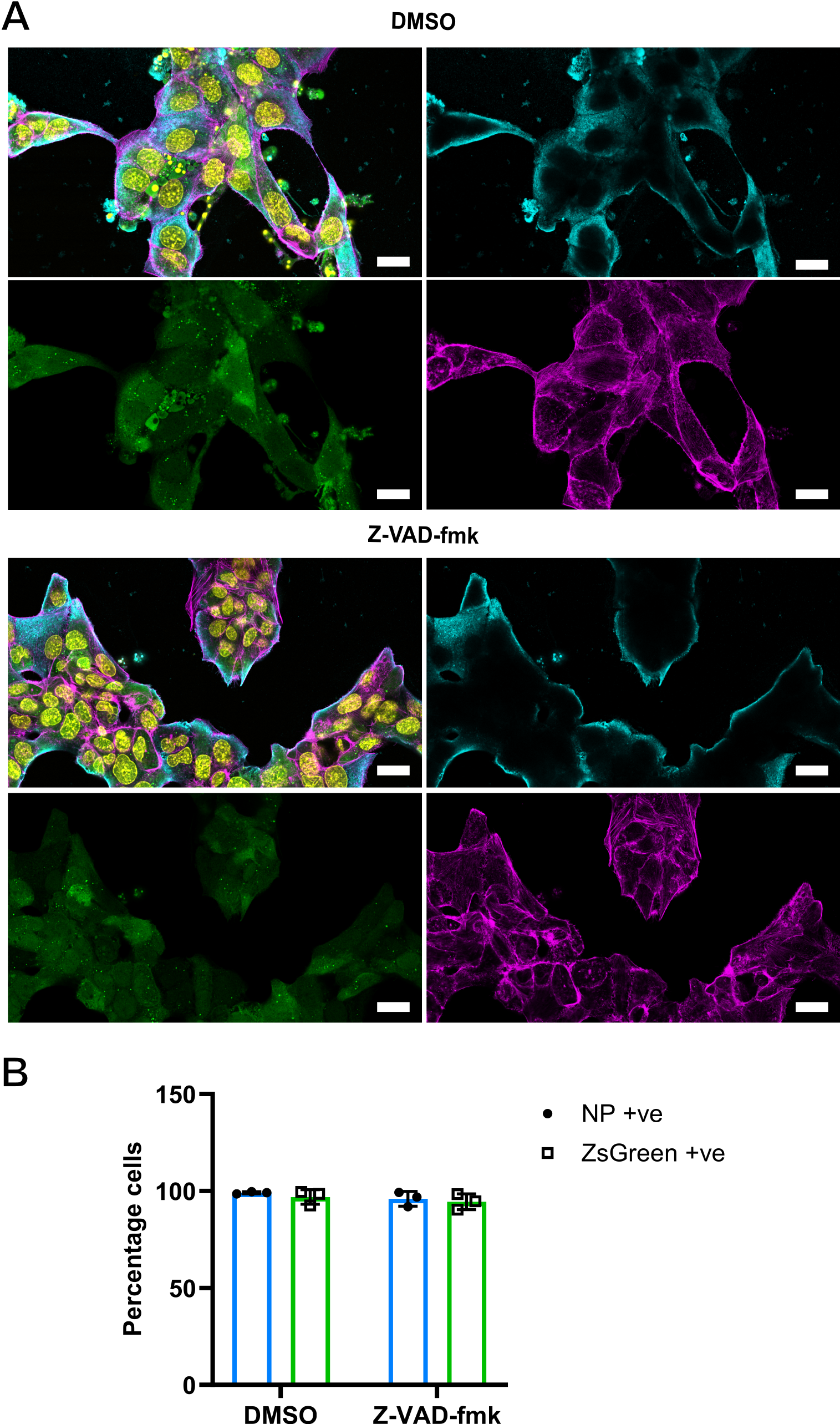

Supplement: S7 Fig — (A) Representative merged and split channel confocal images of BrightFlu infected (MOI 1.5 PFU/cell) MDCK cells 16 hours post infection, treated with either DMSO or 100 µM Z-VAD-fmk 1 hour post infection. Nuclei (yellow), F-actin (magenta), ZsGreen (green), NP (blue). Scale bar = 20 µm. (B) The percentage of cells imaged that are positive for NP or ZsGreen signal following DMSO or 100 µM Z-VAD-fmk treatment. (TIFF) [file ppat.1013191.s007.tiff]

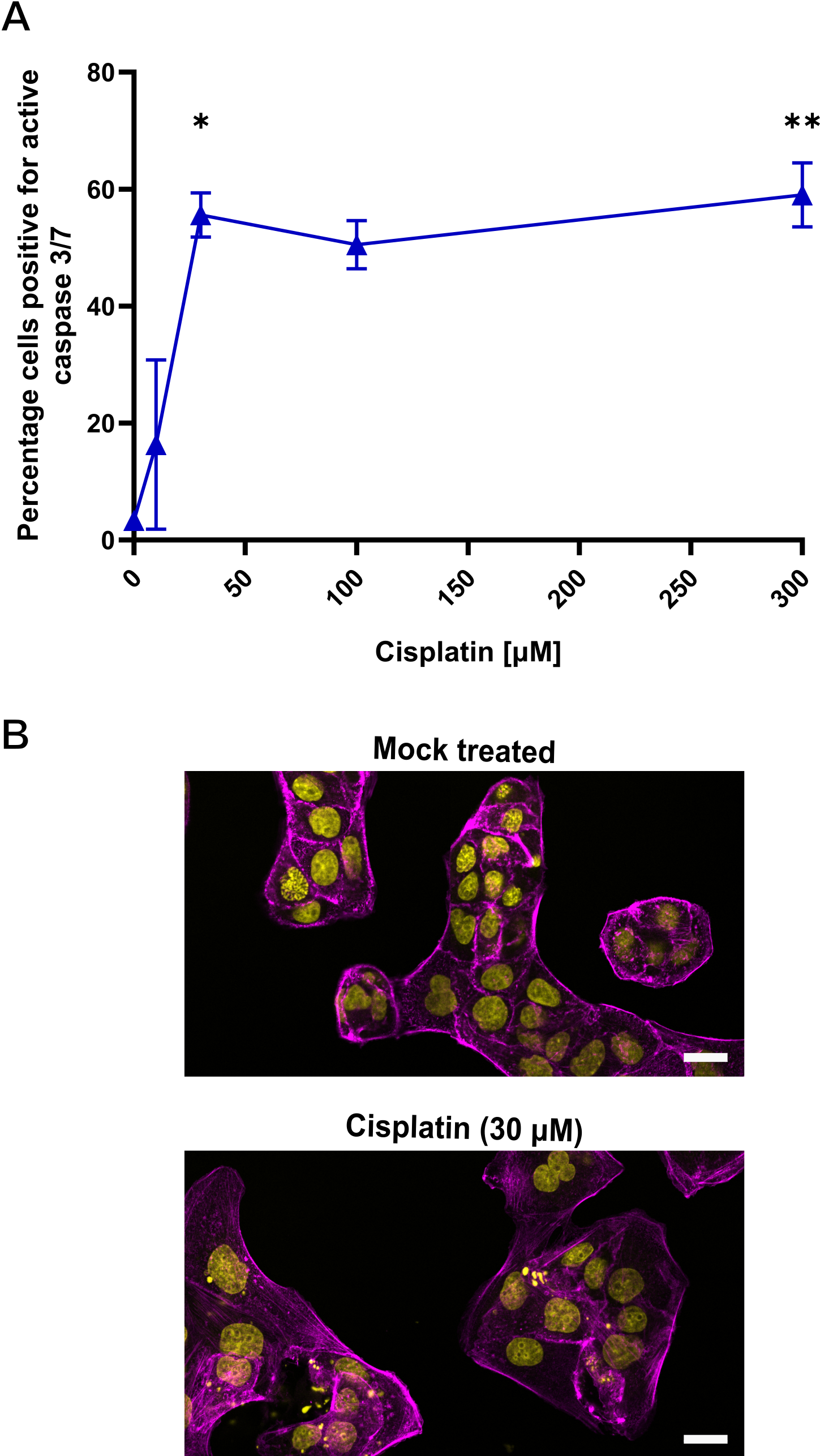

Supplement: S8 Fig — (A) The percentage of cells positive for active caspase 3/7 16 hours post treatment with increasing concentrations of cisplatin. Differences relative to the mock treated control (0 µM) were tested for significance using a Kruskal-Wallis test (n.s. p > 0.05, * p < 0.05). The mean and SD are shown (n = 3). (B) Representative images of 2x tiled super resolution confocal micrographs of MDCKs treated with 30 µM cisplatin or PBS (mock) for 16 hours. Nuclei (yellow), F-actin (magenta). Scale bar = 20 µm. Images are representative of 3 biological replicates. (TIFF) [file ppat.1013191.s008.tiff]

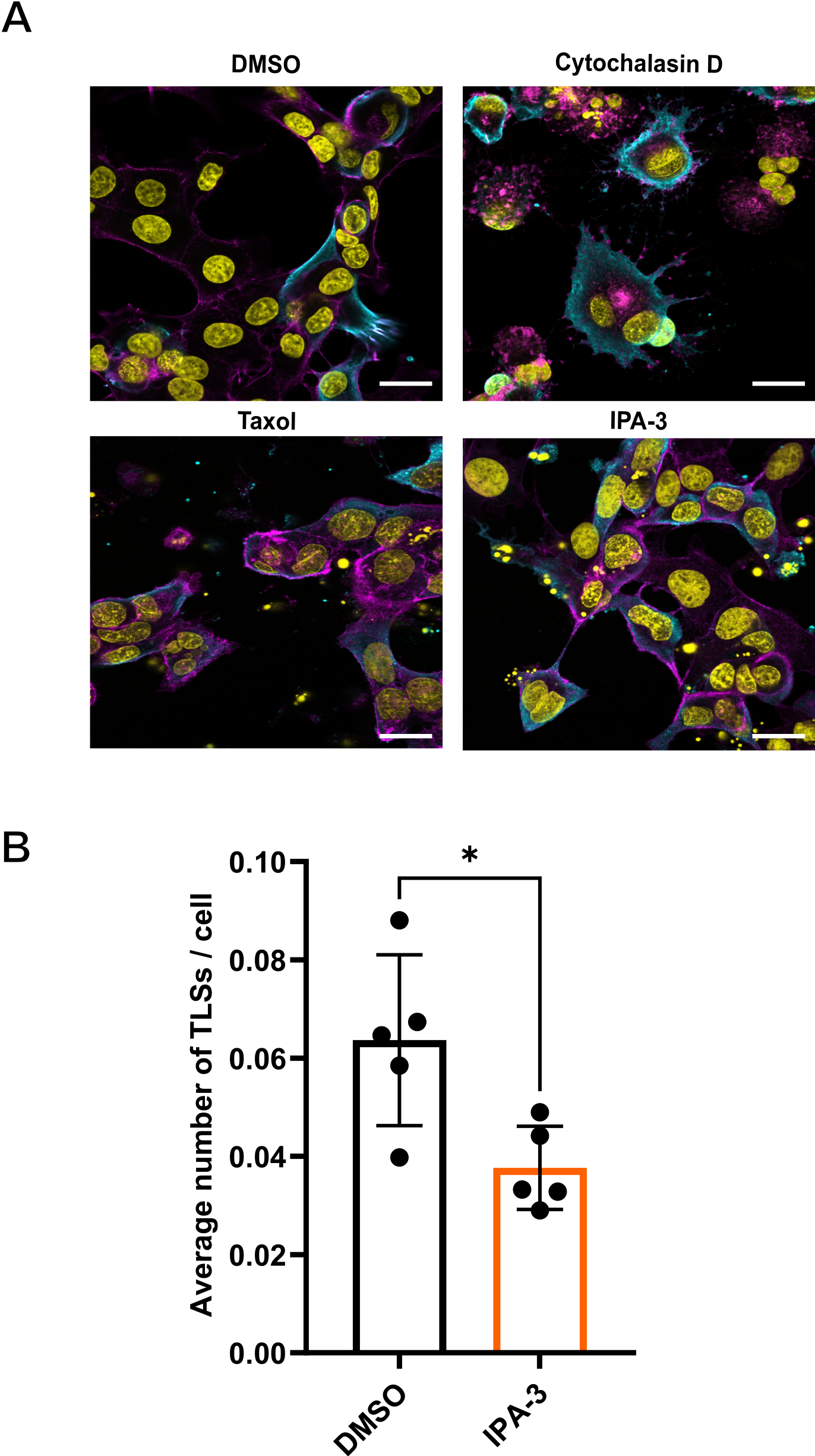

Supplement: S9 Fig — (A) Representative confocal micrographs of MDCKs infected with PR8 at an MOI 0.6, and treated with either DMSO, cytochalasin D (20 µM), taxol (100 µM), and IPA-3 (3 µM) 1 hour post infection. Cells were fixed and immunostained for NP 16 hours post infection. Nuclei (yellow), F-actin (magenta), NP (blue). Scale bar = 20 µm. (B) Average number of TLSs per MDCK cell 16 hours post infection with PR8 at an MOI of 1.5 PFU/cell, either in the presence of DMSO or IPA-3 (3 µM) added 1 hour post infection. Differences in TLS induction was tested for significance by Mann-Whitney test (n.s. p > 0.05, *p < 0.05). The mean and SD are shown (n = 5). (TIFF) [file ppat.1013191.s009.tiff]
