# Supplementary material for: Induction of tunnelling nanotube-like structures by influenza A viruses requires the onset of apoptosis
Source: PLoS Pathog. 2025 Jun 5;21(6):e1013191. doi: 10.1371/journal.ppat.1013191 (PMC12169559; doi:10.1371/journal.ppat.1013191)
Supplement: Attachment — Submitted filename: Response to reviewers comments.docx [file ppat.1013191.s011.docx]

We thank the reviewers for their comments on our paper. In response to their feedback we have performed additional experiments and extensively updated the manuscript. We feel that the revised manuscript was significantly strengthened by this, and appreciate the opportunity to strengthen and clarify the arguments of this paper.

**Part II – Major Issues: Key Experiments Required for Acceptance**

*Reviewer #1: Fig 1B:TNTs and TLSs do not adhere to substratum and are most often found above the plane of imaging.*

We thank reviewer 1 for their comments. Reviewer 1 is correct to point out that by strict definition TNTs do not adhere to the substrate. As our *in vitro* assays do not demonstrate their 3D position we have referred to these structures as ‘TNT-like structures’ (TLSs) in line with other studies (1-4). This distinction and justification is now outlined in lines 69-81. Importantly, even though the technical limitations of what we could image meant that we could not include an investigation of all TLSs in 3D space, we would not expect that to affect the comparisons of relative TLS counts under different conditions on which most of our conclusions were based.

*Have these sections been visualized by changing the plane of focus to see if there are TLSs present reaching across the tissue that would not otherwise be visible by standard confocal imaging?*

For our data relating to imaged tissue sections, which reviewer 1 highlights in Fig 1B, we did change the plane of focus as part of Z-stacks, with maximum intensity projections also being performed. This was required to capture projections that are more likely to extend beyond a single plane of focus within a 3D tissue section. This information is now clearly mentioned in the main body of text (line 139-144), to accompany the detail provided in the methods sections (under confocal microscopy).

*Fig 2A: Since the infection was carried out for 16h, I would expect to see the NP as distinct puncta in the cytosol and within the TLSs. As it stands, it is really difficult to make out the punctae in the TLS or otherwise. Perhaps an image at 20um would provide greater resolution.*

We have included an additional set of enlarged images in S2A Fig to make this observation clearer.

*Fig 2B: These data are contradictory to all previous work with IAV and TNTs showing a significant increase in the number of TNTs upon infection. Please explain this discrepancy.*

Reviewer 1 is correct to point out that TLSs are induced upon IAV infection. However, this observation has exclusively been made in studies comparing high MOI infections to uninfected samples (5, 6). The observation in our study that both infected and uninfected cells can produce TLSs equally was made comparing infected and uninfected cells in culture together under a low MOI infection condition, which is a distinct experimental condition to previous reports that quantified TLS induction. To be specific, our experiment (Figure 2) involved infecting at a lower MOI, and then looked at how infection influences the ability of individual infected (approximately 37% of cells, Figure 2D) and uninfected (approximately 63% of cells, Figure 2D) cells to produce a TLS. We have emphasised and clarified this point in lines 223-235. In the subsequent figures of our study where we investigate TNT induction we use a high MOI infection.

For this reason, we do not believe our data to be contradictory to published literature. Importantly, although Kumar *et al.* were able to show uninfected cells producing TLSs that connect IAV infected cells (7), they did not quantify the frequency of this, and our data provides a novel quantitation of these connections. We included this discussion of the work by Kumar *et al.* in lines 214-220 to address this comment. Our novel work revealed that under infection conditions where not every cell is infected, both infected and uninfected cells can produce TLSs. We have noted in the manuscript that our results, which show that both infected and uninfected cells can produce equal numbers of TLSs in low MOI conditions, are in line with studies of different viruses (lines 592-595) (8, 9).

*It is unclear what are the authors considering as TLS. There is a difference between intercellular connections like filopodial bridges and TNTs in both composition, structure and function, which could maybe account for the discrepancy in the number of TLSs in uninfected and infected cells.*

In response to the reviewer requesting a clearer definition of a TLS, we have clarified this at lines 69-81, and 883-893. We used the criteria of continuous F-actin content and a 5 µm minimum length for classifying TLSs, in part because this reduces the likelihood that the structures we observed are ‘illegitimate’ TLSs, which either lack this cytoskeletal component (such as nanopodia) or rarely extend to such lengths as this (such as filopodia). This is in line with the findings and criteria applied in other studies (6, 10).

*Line 179-181: The authors conclude that the infection status of the A549 cells had no impact on the ability to form a TLS. These conclusions are contrary to previous observations by Kumar et al, Roberts et al and Ganti et al. and need a valid reason as to why.*

We respectfully disagree with the reviewer here. As explained above, our data comparing infected and uninfected cells in the same low MOI infection are not contradictory to previous reports, which compared separate high MOI infection samples and mock-infected samples (5, 6). The ability of both infected and uninfected cells to form TLSs has been seen previously (7) but the quantification of this that we performed here is novel.

*Could it be due to the modification of the membrane composition with GFP? There are less invasive ways to differentially label membranes (DiO dyes etc) that would not alter membrane composition and potentially its function. What was the rationale for using the AcGFP other than retaining the label for multiple passages?*

This is a good point, and we shared the reviewer’s concern about lipid modification when designing this experiment. We therefore assessed the effect of AcGFP membrane labelling, and found that approximately 50% of TLSs were AcGFP positive which was expected given that 50% of cells in the co-culture were AcGFP labelled (supplementary figure S2B). In other words, this modification had no influence on the propensity of modified cells to produce TLSs. We had also attempted to use DiO and DiD dyes, but found that this approach led to significant intracellular staining, loss of signal and passive transfer of dyes between cells, which ultimately made determining the cellular origin and receipt of a TLS impossible. A similar method, using the same lentivirus approach as we applied here, was used previously by Andresen *et al*., who made similar observations regarding the ability of uninfected and infected cells to produce TLSs following HTLV-1 infection (as mentioned in line 201-202) (8). We have added more text to explain that this lentivirus system is not the first of its use for the study of TLSs, in lines 218-220.

*Fig 2F: This observation is again contradictory to previous reports that the percentage of TLSs formed between infected cells is greater than those formed between uninfected cells. The authors should address this discrepancy and provide reasoning as to their contradictory data.*

As noted above, our quantitative data comparing infected and uninfected cells in the same sample are distinct from, and so do not contradict, previous studies comparing separate high MOI infected samples with mock infected samples (5, 6).

*Fig 5: These experiments should be conducted in the presence of microtubule and actin polymerization inhibitors to demonstrate the necessity of the actin cytoskeleton and not microtubules in the cell-cell spread of infection, even in the presence of amantadine and zanamivir.*

We thank the reviewer for this suggestion and we have included data on the frequency of direct cell to cell spread of IAV in the presence of cytochalasin D, IPA-3 and taxol, drugs targeting either actin or microtubules (Fig 8). This new work included both the previously established microplaque assay and another assay using a fluorescent reporter virus and flow cytometry. Our results showed that direct cell to cell spread of IAV infection can be inhibited by TLS inhibiting drugs to different extents, and we suggest that this is dependent on cells’ tolerance of these drugs (i.e. effective concentrations become cytotoxic) as well the cell culture conditions (lines 557-559 and 568- 570). F-actin and microtubule targeting drugs also decreased the frequency of direct IAV cell to cell transfer, consistent with previous studies (5). We have discussed these new findings in the new section “Inhibition of apoptosis reduces the ability of IAVs to directly spread to distant cells” as well as in lines 668-674.

*Since we know that Rab11a is crucial for the trafficking of genomes within TNTs, the authors should look into whether the PR8 and Udorn viruses interact with Rab11a with the same efficacy and speculate on if this could be a rationale for the observed differences between the two viruses.*

The reviewer makes an interesting point, presumably referencing the work from Ganti *et al.* which showed that NP (a proxy for IAV genomes) is only incorporated in <5% of TNTs in the absence of Rab11a (6) and indicating that, Rab11a is essential for the incorporation of NP. However, our data in figure 4, which show a comparable incorporation of NP within TLSs induced by either PR8 or Udorn, suggests that both viruses interact comparably with Rab11a (at least for the direct transfer of viral genomes). The host factors required for the trafficking of IAV through TLSs fall outside the scope of our paper. However, the hypothesis is an interesting one and we have incorporated it into the paper’s discussion (lines 686-691).

*Fig 6: It is nearly impossible to tell which cells are infected and which are not in this figure.*

Split channel images of these micrographs are now included in supplementary figure 7 to more clearly show infection in the presence of DMSO or Z-VAD-fmk.

*Addition of the caspase inhibitor at the time of infection may have significant downstream effects on various other signalling pathways that may affect both viral replication and the induction of TLSs. We do not yet know the mechanisms of TNT and TLS formation and how any perturbation of the apoptosis or any other signalling affects these pathways. Inhibition of caspase3 is known to reduce viral replication which may be why there are fewer TLSs and not necessarily due to the inhibition of apoptosis itself.*

We found that treating infected cells, post adsorption, with Z-VAD-fmk had no antiviral effects. This is shown in supplementary figure 7B which shows comparable levels of infection between drug treated and DMSO treated groups. Furthermore, we show in figure 7B that there is no decrease in the total percentage of cells expressing the fluorophore of a reporter virus (yellow and green data points) when concentrations of Z-VAD-fmk were increased. These experiments demonstrate that the concentrations of Z-VAD-fmk we used do not prevent viral replication at the time points used in this study. Therefore, we believe that our data does indicate that TLS induction during IAV replication directly requires apoptosis, rather than just indirectly needing this as a requirement for IAV replication. Our conclusion is further supported by additional experiments which show that TLS induction only occurs when high levels of infection is accompanied with the onset of apoptosis (Fig 6 and Fig 7D).

*Use of a reversible caspase inhibitor such as Rac-M826 or similar and the use of a positive control for caspase activation such as cisplatin would be able to demonstrate whether simply the inhibition or induction of apoptosis was sufficient to modulate TLS formation. Therefore, the conclusion drawn here is not supported by the data.*

We thank the reviewer for these suggestions. Regarding reversible caspase inhibitors, the fragility of TLSs makes this challenging to implement in practice. The addition of an inducible or reversible caspase inhibitor would likely disrupt TLSs due to the need to remove the plate from the incubator and modify the overlay. This action would be expected to disrupt TLSs and it is unclear how long it would take for TLSs to reform in this scenario. However, the reviewer’s point is an important one and we were keen to address it. To do this, we have included new data from a time course of infection, in which we show that in the absence of a caspase inhibitor, time points that have comparable levels of infection, but differ in the activation of caspases, also differ in the ability of TLSs to be induced, as predicted by our original hypothesis (Fig 6). This experiment allowed us to investigate whether the activation of apoptosis was required for TLS induction without adding caspase inhibitors or movement of the sample during the course of infection.

The reviewer’s suggestion of using cisplatin was extremely helpful. We have included data from this experiment in Fig 7D. We found that cisplatin treatment itself does not significantly increase the average number of TLSs/cell, but when administered to infected cells it causes TLSs to be induced at an earlier time point (Fig 7D). This is an important result which has allowed us to refine our conclusions about the interaction between influenza virus infection and the induction of apoptosis in TLS formation, and we are grateful to the reviewer for suggesting this. We have discussed this result and its implications in lines 494-513 and 647-659.

*Reviewer #2: 1. Figure 1 and SF 1. In a brilliant technical tour-de-force, the authors successfully visualize protruding tubular structures in IAV-infected cells in the lung of an experimentally infected mice.*

We thank reviewer 2 for their description of this work as ‘a brilliant technical tour-de-force,’ and for their helpful suggestions below.

*However, some additional information is needed to contextualize these findings. A) Line 115 states that “TLS appear to have formed in the spaces between cells of the epithelium”. Which respiratory epithelium are we looking at? Pseudostratified columnal ciliar, simpler columnar, cuboidal or squamous? In other words, are we looking at the upper or lower respiratory tract? Please clarify and indicate in which type of epithelial cells IAV PR8 replication is expected/observed here. The statement in. line 125 refers to a “higher density” tissue. Please explain.*

We have added the above requested details in lines 146-149 and 160-163, in particular we have clarified that this is likely pseudostratified columnar epithelial cells of the lower respiratory tract, and is infected by IAVs as expected .

*B) Frequency of tubular protrusion occurrence. Figure 1B shows a picture-perfect protrusion from one cell, Figure 1C shows 3 infected cells of which two are linked by one protrusion and SF1 shows one more example of protrusion. However, how often do these connection to distal cells occur? How many cells did the authors look at to find the three fields of view shown?*

We thank the reviewer for their kind description of our images of TLSs *in vivo*. These were presented as qualitative observations as we were unable to quantify their frequency between cells. This was partly due to the inability to get DAPI staining to reliably work within the tissues without compromising the integrity of thin tissue sections and potentially damaging TLSs. Consequently, we were unable to establish the number of cells in each field of view. In addition, the reporter system only allows us to analyse the form of mG positive cells, and these were sparse and unevenly distributed across tissue sections – perhaps due to the complex anatomy of the infected lung, and the variability of virus deposition across the sites of the lung. The outcome was that connections between mG positive cells were only infrequently observed, a point we have explained in lines 166-172, and that we did not have appropriate comparators to be able to perform any quantitative analysis in this experiment.

*2. Figure 1B. The authors infect MDCK cells in vitro and show two examples of tunneling nanotube-like (TNL) formation that, to my perception, are nor particularly convincing. Perhaps infection of well-differentiated primary respiratory epithelium (upper or lower respiratory tract as appropriate) would yield more convincing data.*

We did consider this point, but, well-differentiated primary respiratory epithelium could not be manipulated at the scale needed for the quantitative observations of TLS required in the *in vitro* portions of our study. This is why we used cancer cell lines, which have a long history of being used for the study of TLS formation, including those that form in response to *in vitro* infections (5, 11). In these cells we observed TLSs that resembled those reported by other studies, including Roberts *et al.* and Ganti *et al.* (5, 6). We defined TLSs by objective criteria, which as shown in Figure 1D (referenced by this reviewer as Figure 1B), match those conventionally used for TLS classification: these structures are F-actin rich, appear to connect distant cells, are longer than 5 µm, and (in the case of infected cells) contain NP.

*3. In Figure 3 MDCK cells were infected with the IAV Udorn strain. Were similar experiments performed with the PR8 strain used for the experiments of Figures 1 and 2, and if yes, were similar results obtained? I wonder why a different strain was used in these experiments, adding a variable.*

In figure 1 we used a PR8 system, which has been extensively used in experimental infections of mice. Figure 2 relates to the ability of both infected and uninfected cells to produce a TLS, as inspired by questions generated from the system used figure 1B and C, which was only able to show TLSs originating from the infected cell (cell morphology was only made visible by mG positive membranes, i.e. infected cells). Therefore, we used a PR8 virus again to infect an airway epithelial cell line (figure 2) for consistency between these systems. Importantly, figures 1 and 2 do not involve any investigation on the induction of TLSs, and so are self-contained within this paper.

In figure 3 we move on to studying the induction of TLSs. In response to the reviewer’s comment we have more clearly signposted the transition of ideas in lines 256-269, and at this point we introduce Udorn.

Roberts *et al.* first reported TLS induction using the Udorn strain (5), therefore, in earnest we transitioned to using this same stain when we began investigating this induction. We have noted this in the text in lines 268-274. Importantly, we also compared the ability of PR8 and Udorn to induce TLSs and incorporate NP within them, as shown in figure 4. As discussed in the text, we did hypothesise that the two strains might induce TLSs differently, but in fact, we found no significant difference between them. Therefore, we do not think that the selection of a single strain when comparing TLS induction to mock infected or treated samples is limiting or causing variability in the findings of our study.

*4. In the text PR8 is referred as a virus making secreted particles and Udorn at a virus making filamentous particles. This simplification is not appropriate because the plots of Figure 4B clearly show that PR8 does make some filaments. This has implications for the Results and the Discussion: the authors cannot state that TLS induction is independent from virion morphology*

We thank the reviewer for their comment. We have implemented the necessary changes throughout including lines 334-337, 348-350, and 403-405.

*5. The last Figure (#6) presents the most significant new observation, which is reported in the title: TNT-like structures do not form when apoptosis is inhibited. However, there is little development of this observation. This could be remediated by testing one or more of the mechanistic hypotheses considered in the Discussion.*

This is a fair point regarding the balance of the paper. While mechanistic hypotheses falls beyond the scope of this paper, we have extended our observations of the importance of apoptosis in the induction of TLSs by IAVs. This includes a time course of infection to measure the kinetics of caspase activation and TLS induction (Figure 6). We have also included experiments with cisplatin, which induces apoptosis and revealed that although apoptosis is necessary for TLS induction it is not sufficient, as virus replication is also required (Figure 7). Finally, and importantly for the relevance of our findings to infection biology, we show that inhibition of apoptosis reduces the direct cell to cell spread of IAV infection between subconfluent cells (Fig 8), indicating a functional role for apoptosis in this process.

*6. The title needs attention. The authors use “requires” (the onset of apoptosis) in the Abstract, which is better than “through” (the onset of apoptosis).*

We thank reviewer 2 for this suggestion. The title has been changed to include “requires” the onset of apoptosis.

*Reviewer #3: Major points:
1. The data in Fig. 6 show that Z-VAD inhibits TLS induction. The critical experiment to test if Z-VAD inhibits cell-cell transmission was not performed.*

We thank reviewer 3 for their comments. We have included new data from experiments that measure the ability of IAVs to undergo direct cell to cell spread in the presence of Z-VAD-fmk and show that, as we would have predicted, this drug significantly reduces the direct cell to cell spread of IAV infection between subconfluent cells (Fig 8).

*Do the kinetics of caspase activation/apoptosis during infection correlate with the kinetics of induction of TLS? There does not appear to be anything conclusive here.*

We thank this reviewer for their suggestion and we have performed an infection time course to measure the kinetics of caspase activation and TLS induction (Figure 6). This revealed that TLSs are induced by 16 hours post infection, an infection time point that also see the activation of caspases. However, TLSs are not induced at 12 hours post infection, prior to caspase activation, despite comparable levels of infection (Figure 6). Therefore, this reveals that the onset of infection induced apoptosis correlates with the onset of apoptosis, as detailed in lines 455-470.

*2. The authors present a microplaque assay to quantitate cell-cell transmission. However, there is no evaluation of the presence of TLS in the cells of the microplaque, making it difficult to conclude if this transmission is mediated by TLS. There is no test of the effect of Z-VAD in this assay.*

We thank the reviewer for their comment, however, we and others have been unable to visualise TLSs that connect cells over a distance in the microplaque assay due to the required high cell density of this assay. This has resulted in the dependency of TLS inhibiting drugs within the microplaque assay overlay to ascertain whether TLSs are present and functioning to traffic infection, as indicated by a reduction in microplaque formation when these drugs are included (5).

To address this comment we have included data from a microplaque assay that include drugs that inhibit TLS formation (5), including Z-VAD-fmk (Figure 7C & 8). Drugs were selected for their differences in mechanisms of action and cytoskeletal targets (as explained in lines 522-527).

We found that these drugs do not have an inhibitory effect of the direct cell to cell spread of IAV infection in the microplaque assay. However, given that it is not possible to visualise and verify that TLSs connect cells in a confluent monolayer (as explained in lines 547-549), we implemented another assay with subconfluent cells which was conducive to TLS formation and observation. This revealed that drugs inhibiting TLSs (including Z-VAD-fmk) do also reduce the frequency of direct cell to cell spread (Figure 8), as we would have predicted.

*It is also puzzling that the 2 positive microplaques shown in Fig. 5A are quantitated similarly even though the one on the right has only a tiny part of an adjacent cell that appears positive. How is the area of the plaque accurately determined in this assay?*

Reviewer 3 is correct to highlight that the two positive microplaques indicate two regions where two or more adjacent cells are NP positive. These regions meet the threshold of a microplaque classification because there are two adjacent cells positive for NP. Similar classification has been used by Brooke et al. to distinguish between semi infectious particles and fully infectious particles (12). Therefore, this type of analysis has become well established and has subsequently become a feature of the Nexcelom Celigo imaging cytometer’s software. This analysis applies a threshold in fluorescent area that was set to be greater than the average area of single NP positive cell. The Celigo analysis then measures the unique area of all fluorescent regions that either exceed this thresholded area (indicating a microplaque) or are smaller than it (indicating s single NP positive cell). The subsequent mean area of only those fluorescent areas greater than a single cell was plotted in figure 5D and F. Therefore, whilst both microplaques in this image would be scored as individual microplaques, they are recorded differently in terms of their area. Details of the analysis are provided in the methods section under Image Analysis, and to clarify this for the reader we have included additional information about how this gating threshold was established and optimised on the machine (lines 895-900).

*3. The results in Fig. 2 vs. Fig. 3 are confusing. Fig. 2 shows that both infected and uninfected cells can make TLS, and if anything the uninfected cells have a higher proportion of cells making them. Fig. 3 shows that only infected cells make TLS. The numbers of TLS are also very low in Fig 3, ~0.02 TLS/cell in Fig. 3C. It is very difficult to interpret these two apparently contradictory figures.*

We thank the reviewer for raising this point. Although, the results of the two figures differ, they are not in fact contradictory, as figures 2 and 3 show data from experiments with different infection conditions. In Figure 2 all cells we examined had been exposed to a low MOI of virus, and we then explored whether infection status of individual cells influenced TLS origin or receipt. In Figure 3, we compared separate samples, one in which was infected at all and another in which cells were all infected at a high MOI.

Furthermore, in each experiment, different cell types were selected in each figure, to reflect the experimental design. In Figure 2, which followed directly from the *in vivo* studies and which addressed TLS pathfinding, A549s were selected as they are lung epithelial cells which more appropriately addresses the observations we made in terms of TLS origin in the lungs of infected mice (Figure 1), a point that we now explain in lines 188-190 and 214-218. In Figure 3, in which we began to address the induction of TLSs by IAVs, MDCKs were chosen to measure TLS induction following high MOI infection as these cells are highly permissive to infection and have been consistently used to measure TLS induction following high MOI IAV infection, a point explained in lines 268-270.

*4. To this reviewer, the in vivo results in Fig. 1 were unconvincing. A couple of projections from infected cells are visualized, but the paper then drops the system (which seemed like a potentially promising method to identify infected cells in mice). This reviewer was not convinced, given the paucity of data, that these were actually TNLs or that they play a role in vivo.*

The structures we show in this work strongly resemble those previously published in other publications and include notable features such as bulbous termini (discussed in lines 155-158) (13). Therefore, we believe that our observations are on par with previous published findings on TLSs *in vivo*. It is unknown whether any TLS structure observed in tissue to date play a role *in vivo* due to the constraints associated with working with fixed or biopsied tissue. This is a limitation that is currently applicable to the field as a whole.

After validating *in vivo* that these structures are less likely to be an artefact of *in vitro* cell culture, we required a much more flexible, controlled and higher throughput system to uncover how IAVs were triggering the induction of TLSs (we have expanded on this point in lines 174-178). This meant that we had to transition from the *in vivo* system and implement *in vitro* systems similar to those that first reported an induction of TLSs by IAV (5).

*Do uninfected cells make similar projections, as appears to be the case in Fig. 2?*

In our *in vivo* system we are not able to see significant detail of the membranes of uninfected cells within the lung. Only the GFP-labelled membranes of infected cells were made distinctly visible against the membranes of the majority of neighbouring uninfected cells that crowded together in the dense tissue and remain labelled with tdTomato.

**Part III – Minor Issues: Editorial and Data Presentation Modifications**
Please use this section for editorial suggestions as well as relatively minor modifications of existing data that would enhance clarity.

*Reviewer #1: (No Response)*

*Reviewer #2: • Fig. 3B. Indicate which reagents were used to stain cytoplasm and nucleus.*

Details of the reagents used to stain cells is included in the materials and methods section under Immunocytochemistry and immunostaining of virus particles.

*• Line 407: “crowded, mobile environment of the respiratory epithelium”. Which sector of this epithelium? Please articulate.*

We have addressed this in line 583-585, where we now articulate a focus on the pseudostratified respiratory epithelium for which we know and show IAV to infect.

*• Line 331: 25% of infected cells infected their neighbors. Discussion point: how does this compare to the infection with other respiratory viruses? (Relevant data have been obtained in well-differentiated primary human airways epithelial cells).*

We thank the reviewer for this comment and we have addressed this in lines 676-679. We note that our study is focused specifically on the direct cell to cell spread of infection and quantitative data on the frequency of direct cell to cell spread within well-differentiated primary human airway epithelial cell is currently lacking. To our knowledge, only qualitative evidence for this has so far been presented with measles virus (14).

*Reviewer #3: Minor points:
1. It’s unclear why the authors use the term TNT-like structures (TNL) rather than TNT. What are the differences they are highlighting? This should be addressed early on. The authors should more carefully describe how they define a TNL structure in the tissues and cells being assayed. Is a virus marker always used? Actin?? What is the rigorous definition here, especially given that uninfected cells also produce TNLs.*

We thank the reviewer for their comments and we have included extra detail to explain this early in the manuscript in lines 69-81. This extra detail accompanies further information in the methods section (under image analysis) which states that a stain or marker of infection is always included. The lack of an infection marker within a TLS that meets the other criteria is insufficient to classify it as TLS negative; F-actin stain throughout the length of a TLS that connects at least two cells is, however, an essential requirement.

*2. The paper switches cell types between MDCK and A549 (as well as the initial in vivo experiments in FIgu 1). The reasons for this are not clear and should be addressed.*

Similar to our response to the third comment of reviewer 3 (above), we have added a justification and background for the use each cell type found in lines 214-220 and 268-270.

The justification of the transition away from the *in vivo* system is addressed in response to the fourth comment made by reviewer 3, above.

*3. Please add concentrations of reagents and experimental timing to the legends where it is missing. For example, in the flow analysis of Figure 6A it is not clear what concentration of Z-VAD is used or when it was added post-infection.*

We have added information to all figure legends to include these details.

*4. In Fig 6B, the color diagram of the conditions is very confusing and needs clearer explanation.*

Additional clarification has been provided within the figure legend.

5. Line 361: According to the authors’ thesis, amantadine should not inhibit the “transfer” of viruses but rather their uncoating in the target cell. Please reword.

We have changed this accordingly in lines 432-434.

*6. Please clarify in the figures if/when TLS/cell means TLS/infected cell.*

All the figures that quantify the induction of TLS are normalised to all cells. There are no instances where TLS/infected cell is appropriate.

**References for Rebuttal**

1.	Okafo G, Prevedel L, Eugenin E. Tunneling nanotubes (TNT) mediate long-range gap junctional communication: Implications for HIV cell to cell spread. Scientific Reports. 2017;7(1):16660.

2.	Byford O, Shaw A, Tse HN, Moon-Walker A, Saphire E, Whelan S, et al. Lymphocytic choriomeningitis arenavirus utilises intercellular connections for cell to cell spread. Scientific Reports. 2024;14.

3.	Gousset K, Marzo L, Commere PH, Zurzolo C. Myo10 is a key regulator of TNT formation in neuronal cells. J Cell Sci. 2013;126(Pt 19):4424-35.

4.	Gurke S, Barroso JFV, Hodneland E, Bukoreshtliev NV, Schlicker O, Gerdes H-H. Tunneling nanotube (TNT)-like structures facilitate a constitutive, actomyosin-dependent exchange of endocytic organelles between normal rat kidney cells. Experimental Cell Research. 2008;314(20):3669-83.

5.	Roberts KL, Manicassamy B, Lamb RA. Influenza A virus uses intercellular connections to spread to neighboring cells. J Virol. 2015;89(3):1537-49.

6.	Ganti K, Han J, Manicassamy B, Lowen AC. Rab11a mediates cell-cell spread and reassortment of influenza A virus genomes via tunneling nanotubes. PLoS Pathog. 2021;17(9):e1009321.

7.	Kumar A, Kim JH, Ranjan P, Metcalfe MG, Cao W, Mishina M, et al. Influenza virus exploits tunneling nanotubes for cell-to-cell spread. Scientific Reports. 2017;7(1):40360.

8.	Omsland M, Pise-Masison C, Fujikawa D, Galli V, Fenizia C, Parks RW, et al. Inhibition of Tunneling Nanotube (TNT) Formation and Human T-cell Leukemia Virus Type 1 (HTLV-1) Transmission by Cytarabine. Scientific Reports. 2018;8(1):11118.

9.	Sherer NM, Lehmann MJ, Jimenez-Soto LF, Horensavitz C, Pypaert M, Mothes W. Retroviruses can establish filopodial bridges for efficient cell-to-cell transmission. Nature cell biology. 2007;9(3):310-5.

10.	Henderson JM, Ljubojevic N, Belian S, Chaze T, Castaneda D, Battistella A, et al. Tunnelling nanotube formation is driven by Eps8/IRSp53‐dependent linear actin polymerization. The EMBO Journal. 2023;42(24):e113761.

11.	Rustom A, Saffrich R, Markovic I, Walther P, Gerdes H-H. Nanotubular highways for intercellular organelle transport. Science. 2004;303(5660):1007-10.

12.	Brooke CB, Ince WL, Wrammert J, Ahmed R, Wilson PC, Bennink JR, Yewdell JW. Most influenza a virions fail to express at least one essential viral protein. Journal of virology. 2013;87(6):3155-62.

13.	Chinnery HR, Pearlman E, McMenamin PG. Cutting edge: Membrane nanotubes in vivo: a feature of MHC class II+ cells in the mouse cornea. J Immunol. 2008;180(9):5779-83.

14.	Singh Brajesh K, Li N, Mark Anna C, Mateo M, Cattaneo R, Sinn Patrick L. Cell-to-Cell Contact and Nectin-4 Govern Spread of Measles Virus from Primary Human Myeloid Cells to Primary Human Airway Epithelial Cells. Journal of Virology. 2016;90(15):6808-17.
